# Assessing the oral health of an ageing population: methods, challenges and predictors of survey participation

**DOI:** 10.1111/j.1741-2358.2011.00540.x

**Published:** 2012-06

**Authors:** Debora C Matthews, Martha G S Brillant, Joanne B Clovis, Mary E McNally, Mark J Filiaggi, Robert D Kotzer, Herenia P Lawrence

**Affiliations:** 1 Department of Dental Clinical Sciences, Faculty of Dentistry, Dalhousie University HalifaxNS, Canada;; 2 School of Dental Hygiene, Faculty of Dentistry, Dalhousie UniversityHalifax, NS, Canada;; 3 Department of Applied Oral Sciences, Faculty of Dentistry, Dalhousie UniversityHalifax, NS, Canada;; 4 Discipline of Dental Public Health, Department of Biological and Diagnostic Sciences, Faculty of Dentistry, University of TorontoToronto, ON, Canada

**Keywords:** oral health, surveillance, ageing, survey participation

## Abstract

**Assessing the oral health of an ageing population: methods, challenges and predictors of survey participation:**

**Objectives:**

To examine predictors of participation and to describe the methodological considerations of conducting a two-stage population-based oral health survey.

**Methods:**

An observational, cross-sectional survey (telephone interview and clinical oral examination) of community-dwelling adults aged 45–64 and ≥65 living in Nova Scotia, Canada was conducted.

**Results:**

The survey response rate was 21% for the interview and 13.5% for the examination. A total of 1141 participants completed one or both components of the survey. Both age groups had higher levels of education than the target population; the age 45–64 sample also had a higher proportion of females and lower levels of employment than the target population. Completers (participants who completed interview and examination) were compared with partial completers (who completed only the interview), and stepwise logistic regression was performed to examine predictors of completion. Identified predictors were as follows: not working, post-secondary education and frequent dental visits.

**Conclusion:**

Recruitment, communications and logistics present challenges in conducting a province-wide survey. Identification of employment, education and dental visit frequency as predictors of survey participation provide insight into possible non-response bias and suggest potential for underestimation of oral disease prevalence in this and similar surveys. This potential must be considered in analysis and in future recruitment strategies.

## Introduction

Canadians are living longer^1^,^2^ and retaining more of their natural teeth than in previous generations, emphasising a need for greater understanding of oral health throughout the lifespan. The questions regarding appropriate interventions for older adults are complicated by the unknown impact of the ageing baby-boomer generation on the need and demand for oral care. Health systems policy planners in Canada must be aware of, and prepared for, the variety of conditions and challenges that will be posed by this population.

A clear understanding of baseline oral health status and treatment needs is essential to establish oral health priorities that will contribute positively to health throughout the lifespan. Without these basic data, it is difficult to answer complex research questions, to determine how and where to direct education and treatment interventions, or to affect policy change. Until recently, there was little data to reflect the oral health status or treatment needs of Canadians. Statistics Canada recently completed the Canadian Health Measures Survey (CHMS), a national health status survey that included an oral health component^3^. However, the CHMS was not designed to assess oral health status at the provincial level and did not sample populations from all provinces. It is at the level of provincial governments that oral health policy decisions are normally made in Canada.

This paper describes a survey designed to assess the oral health status of adults age 45 and older living in Nova Scotia, Canada. In the province of Nova Scotia, 15.4% of the population is age 65 or older, representing the oldest provincial population in Canada^1^. Compared with other locations in Canada, the relatively small geographical span of Nova Scotia makes the entire population accessible to surveying with minimal expense and travel. The objectives of this paper are to examine predictors of participation in an oral health survey involving a clinical oral examination and to describe the methodological considerations and challenges in conducting a population-based oral health survey on adults aged 45 and older in urban and rural Canada.

## Methods

Based on data from a pilot study^4^, we conducted an observational, cross-sectional survey of adults aged 45–64 and aged 65 and older, living in Nova Scotia. The sample consisted of individuals living independently in rural and urban settings. Residents of long-term care facilities were also surveyed, but are not included in this paper. Only participants capable of giving informed consent, in writing or verbally, were included. Individuals with cognitive impairment were excluded from the study.

The oral health assessment measured four principle components: the impact of oral health on quality of life, utilisation of oral health care services, access to oral health care and clinical oral health status. The first three components were measured through a questionnaire format using a telephone survey. The last was measured by clinical examination consisting of an intraoral examination only; no radiographs were taken. Ethics approval was received from the Health Sciences Research Ethics Board at Dalhousie University and District Health Authorities where required.

### Sample size determination

The minimal sample size required was determined using the following formula: 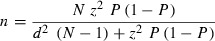


where *n* = sample size with finite population correction, *N* = population size, *P* = expected prevalence, *d* = desired precision, and *z* = value from normal distribution for the desired confidence level (95%; z = 1.96).

The population size [231 941 people aged 45–64 years and 133 571 aged 65 years and older (total = 365 512)] was known from the 2006 census for Nova Scotia^5^. The expected population prevalence rate of oral disease was taken from an American survey of 5603 adults aged 40 and more (53%: periodontal disease, aged 65+)^6^. Using a confidence level of 95% and an error rate of 5%, the calculated sample size was 764 participants [382 ‘seniors’ (≥65 years) and 382 ‘pre-seniors’ (45–65 years)].

### Sampling frame and sample selection

Participants were recruited and interviewed by a contracted telemarketing company. Communities to be surveyed were selected to correspond to the locations of a random selection of all long-term care facilities in Nova Scotia, Canada (prepared for the long-term care component of this survey, which is not described here). For each community, a call list targeting adults 45 years and older living within 20 km of the community was obtained from a commercial call-list supplier. Telephone numbers were selected at random from each call list and were then called up to three times or until contact was made. Eligible adults were asked to take part in both a telephone survey and clinical examination. All those who consented to both were interviewed and given appointments for a clinical examination within two weeks at a location in their community. The software Appointment Quest® (Appointment Quest LLC, Broomfield, CO, USA) was used by the survey company to book appointments at each of the geographical sites visited. To compensate for no-shows, approximately one-third of the appointments for a given day were double-booked. Participants were given a toll-free telephone number to contact the research coordinator with questions about the survey or to cancel appointments.

As an incentive to participate, all participants who completed the study were entered into a draw for one of two prizes of $250 (CAD).

### Questionnaire

Oral Health–Related Quality of Life (OHQoL) was measured using the OHIP-14 questionnaire^7^,^8^– a self-report questionnaire consisting of 14 questions under three domains: *physical function*, including eating, speech and swallowing; *psychosocial function*, including worry or concern about oral health, self image, self-consciousness about oral health and avoidance of social contacts because of oral problems; and *pain or discomfort*.

In addition to the OHIP-14, participants were asked a number of questions derived from the 2008 CHMS^9^. These included demographics; dental care utilisation, including access to dental insurance and frequency of visiting a dental professional; perceived general health and oral health; regular oral hygiene habits; presence of chronic health conditions; medication use; smoking and alcohol history; sun exposure; and income. Additional survey questions determined the amount paid for dental care; employment status; and whether the individual had avoided dental treatment. The entire questionnaire was translated into French and reviewed by an academic fluent in the Acadian dialect. The interview took an average of 26.3 ± 6.5 min (range 11.3–50.9) to complete.

### Clinical examination

The clinical examination was based on WHO criteria modified for the Oral Health Module of the CHMS carried out in Canada from 2007 to 2009^10^. It was customised for our survey of adults in that determination of presence and degree of fluorosis and orthodontic skeletal classifications were removed, and examination of jaw function and prosthetic quality was added. Standard dental operatories were used where available. Otherwise, participants were examined using a portable A-dec® (A-dec Inc., Newberg, OR, USA) chair and an Aseptico® light (Aseptico Inc., Woodinville, WA, USA) or a Mountain Equipment Co-op® headlamp (Mountain Equipment Co-op, Vancouver, BC, Canada). The average duration of the clinical exam was 14.6 ± 5.7 min (range 2.9–40.6).

### Data management and analysis

Telephone interviewers used a script developed for the study, and interview data were entered directly into an electronic database. Frequent teleconferencing between the researchers and telephone company managers addressed concerns or questions raised by interviewees. Audiotapes of interviews were reviewed to identify errors in content and to improve presentation style and audio modulation of those conducting the telephone interviews. The same telephone interviewers were used throughout the data collection period.

All clinical data were directly entered into a password-protected database on a web-based platform, by a single dedicated research assistant. Where no internet connection was available, the data were stored on a laptop.

Survey outcome rates (cooperation and response rates) were calculated using the American Association for Public Opinion Research (AAPOR) standard definitions of dispositions and response rate calculator^11^. Statistical tests were performed using PASW Statistics 17 (IBM®; SPSS Inc.®, Chicago, IL, USA).

Chi-square tests were used to compare the socio-demographic characteristics of the target and study populations. To determine which factors might influence a participant’s likelihood to follow through in attending a clinical examination after completing a phone interview, those community participants who completed both the interview and the clinical examination (completers) were compared with those who only completed the interview (partial completers). Bivariate analysis (chi-square tests, the two-independent samples *t*-test) was used to compare the two groups on socio-demographic variables as well as self-perceived general health status, oral health status and use of oral health care services. Forward stepwise logistic regression (using the Wald statistic as the addition criteria; 0 = completer, 1 = partial completer) was performed to analyse for predictors of survey completion. Statistical tests were two-tailed and interpreted at the 5% significance level.

## Results

### Survey response

**Figure 1:**
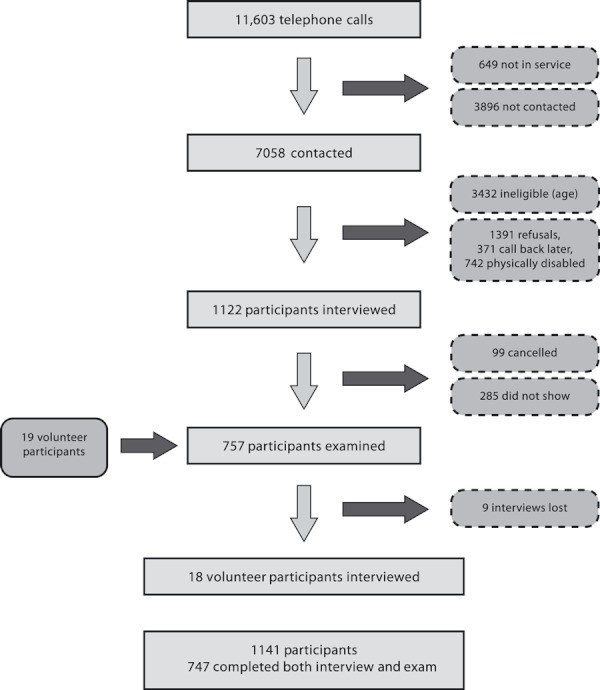
Flow diagram of participant recruitment.

Surveys were conducted in 22 communities from October to November 2008 and April to October 2009. In all, 11 603 phone numbers were called; 61% made contact with a person (Fig. 1). Of those contacted, 16% completed the interview. Forty-nine per cent were ineligible because of age, 20% declined to participate, 5% asked to be called back another time and 10% said they were physically disabled, and therefore unable to participate. The resulting cooperation rate (AAPOR Cooperation Rate 1 = number who completed/number contacted who were eligible) for the interview component of the survey was 35%. The response rate (AAPOR Response Rate 3 = number who completed/estimated number of eligible cases in the sample) for the interview was 21%. In calculating the response rate, the assumption was made that the proportion of eligible cases was the same for both the contacted and non-contacted sample^11^.

All those interviewed were given an appointment for a clinical examination. Sixty-six per cent (738) of these attended and completed the examination; the remainder either cancelled their appointment (9%) or failed to show (25%). The resulting cooperation rate (AAPOR Cooperation Rate 1) for the examination component of the survey was 23%, and the response rate was 13.5% of all eligible cases (AAPOR Response Rate 3). An additional 19 people volunteered to have a clinical examination (usually because they were accompanying someone else to their examination), and 18 of these subsequently completed the phone interview. Interview data for nine participants were lost by the telephone marketing company.

In total, there were 1141 community participants, including 747 ‘completers’ for whom we have both interview and clinical examination data, 384 ‘partial completers’ for whom we have only interview data and 10 for whom we have only clinical data (the last are not included in these analyses). Of participants who completed the clinical examination, the study exceeded the 45–64-age-group target sample size (411/382) and recruited 88% of the ≥65-age-group target sample size (336/382).

### Study versus target populations

Data on the target populations (adults aged 45–64 and 65 and older, living in Nova Scotia) were obtained from the Statistics Canada 2006 census^12^–^17^ (Table 1). The 45–64 study population was comparable to the target 45–64 population with regard to rural and urban distribution, marital status and country of birth. Females were overrepresented in this group compared with the target population (64–65% vs. 51%); the proportion of participants with an education beyond the high school level was higher than the target population (62–66% vs. 56%), and fewer participants were employed than in the target population (44–48% vs. 64%). Median household income for this age group may be slightly higher than the target population; however, this is uncertain, as household income data for individuals aged 45–64 was not available from the 2006 census; median household income for ages 15–64 is reported instead. When a weighting factor is applied to correct for the gender imbalance, it has little effect on the other sample-to-target comparison characteristics.

**Table 1:** Characteristics of the target and study populations.

	Target population^a^		Study population: interview (±examination)^c^		Study population: interview and examination^c^	
			
	45–64 years^b^	≥65 *years*	45–64 years (N = 629)	≥65 years (*N = 502)*	45–64 years (*N* = 411)	≥65 years (*N = 336)*
Gender, *n* (%)						
Female	137 795 (51.3)	78 810 (57.0)	**405** (**64.4**)	306 (61.0)	**266** (**64.7**)	198 (58.9)
Male	130 925 (48.7)	59 420 (43.0)	**224** (**35.6**)	196 (39.0)	**145** (**35.3**)	138 (41.1)
Location, *n* (%)						
Rural	100 875 (37.5)	57 230 (41.4)	255 (40.5)	207 (41.2)	165 (40.1)	146 (43.5)
Urban	167 835 (62.5)	80 980 (58.6)	374 (59.5)	295 (58.8)	246 (59.9)	190 (56.5)
Education, *n* (%)						
Less than high school	61 210 (22.9)	57 325 (43.7)	**179** (**28.5**)	**174** (**34.9**)	**101** (**24.6**)	**103** (**30.7**)
Completed high school	55 170 (20.7)	25 100 (19.2)	**60** (**9.6**)	**65** (**13.0**)	**38** (**9.3**)	**39** (**11.6**)
Post-secondary	150 575 (56.4)	48 640 (37.1)	**389** (**61.9**) missing, *n =* 1	**260** (**52.1**) missing, *n =* 3	**271** (**66.1**) missing, *n =* 1	**193** (**57.6**) missing, *n =* 1
Marital status, *n* (%)						
Married	211 376 (77.1)	79 853 (57.1)	498 (79.2)	306 (61.0)	331 (80.5)	**217** (**64.6**)
Widowed	8738 (3.2)	42 372 (30.6)	23 (3.7)	133 (26.5)	15 (3.6)	**77** (**22.9**)
Divorced	27 286 (10.0)	8889 (6.4)	43 (6.8)	33 (6.6)	28 (6.8)	**18** (**5.4**)
Single, never married	26 628 (9.7)	8154 (5.9)	65 (10.3)	30 (6.0)	37 (9.0)	**24** (**7.1**)
Country of birth, *n* (%)						
Canada	249 375 (93.4)	119 825 (91.4)	578 (92.0)	**440** (**88.2**)	378 (92.2)	**291** (**87.1**)
Other	17 585 (6.6)	11 245 (8.6)	50 (8.0) missing, *n =* 1	**59** (**11.8**) missing, *n =* 3	32 (7.8) missing, *n =* 1	**43** (**12.9**) missing, *n =* 2
Employment, *n* (%)						
Full-time/Part- time	169 285 (63.6)	9445 (7.0)	**299** (**47.7**)	39 (7.8)	**180** (**43.9**)	23 (6.9)
Unemployed	12 745 (4.8)	800 (1.0)	**82** (**13.1**)	5 (1.0)	**55** (**13.4**)	4 (1.2)
Not in labour force	84 130 (31.6)	119 515 (92.0)	**246** (**39.2**) missing, *n =* 2	456 (91.2) missing, *n =* 2	**175** (**42.7**) missing, *n =* 1	308 (91.9) missing, *n =* 1
Median household income						
Sole adult	15–65 years: $25 813 (*n* = 61 995)	$19 202 (*n* = 37 950)	$30 000–$40 000 (*n =* 117; missing, *n =* 32)	$20 000–$30 000 (*n =* 147; missing, *n =* 58)	$30 000–$40 000 (*n =* 74; missing, *n =* 14)	$20 000–$30 000 (*n =* 93; missing, *n =* 33)
Couple	both 15–65 years: $61 795 (*n* = 205 010)one ≥65 years: $49 668 (*n* = 26 895) both ≥65 years: $46 256 (*n* = 30 550)		$60 000–$80 000 (*n =* 374; missing, *n* = 106)	$40 000–$50 000 (*n =* 220; missing, *n =* 77)	$60 000–$80 000 (*n =* 249; missing, *n =* 74)	$40 000–$50 000 (*n =* 162; missing, *n =* 48)

a Statistics Canada 2006 Census of Population – Nova Scotia^12^–^17^.

b Unless otherwise noted.

c Variables in bold are significantly different from the population parameters (Chi-square test at *p* < 0.05).

The 65 and older study population was comparable to the 65 and older target population with regard to gender distribution, rural and urban distribution, employment status and median household income (Table 1). A higher proportion of the 65 and older sample was born outside of Canada than the target population (12–13% vs. 9%), and the proportion with an education beyond the high school level was higher than in the target population (52–58% vs 37%). Within the 65 and older subsample, who completed both the interview and examination, a higher proportion were married than in the target population (65% vs. 57%).

### Completers and partial completers

Completers (participants who completed both the interview and the clinical examination) and partial completers (those who only completed the interview) did not differ on age, gender distribution, rural and urban distribution, language spoken or income. Partial completers were more likely than completers to work, be unmarried, or have a lower level of education (Table 2a). Partial completers also had lower ratings of self-perceived general and oral health and visited their dentist less frequently than full completers (Table 2b). Forward stepwise logistic regression was performed using the significant variables in Table 2a,b to analyse which variables were predictors for completion. This multivariate analysis indicated that participants who did not work were more likely to be completers than those who did work (Adjusted odds ratio = 1.59); those who visited a dentist frequently (once or more per year) were more likely to be completers than those who visited a dentist less than once per year (OR = 1.44); and participants with post-secondary education were more likely to be completers than those with less than a high school education (OR = 1.83) (Table 3).

**Table 2:** Comparison of completers vs. partial completers: (a) socio-demographic characteristics and (b) self-perceived general and oral health status and health services utilisation.

	Completers (*N = 747)*	Partial completers (*N = 384)*	p Value^a^
(a)			
Age (year), mean ± SD^b^ (range)	63.8 ± 10.4 (44–92) (*N =* 726; missing, *n =* 21)	63.2 ± 11.3 (44–92) (*N* = 365; missing, *n =* 58)	0.409^c^
Gender*, n* (%)			
Female	464 (62.1)	247 (64.3)	0.508
Male	283 (37.9)	137 (35.7)	
Location*, n* (%)			
Rural	311 (41.6)	151 (39.3)	0.494
Urban	436 (58.4)	233 (60.7)	
Education*, n* (%)			
Less than high school	204 (27.4)	149 (39.0)	**<0.001**
Completed high school	77 (10.3)	48 (12.6)	
Post-secondary	464 (62.3) missing, *n =* 2	185 (48.4) missing, *n =* 2	
Marital status*, n* (%)			
Married	508 (68.0)	227 (59.1)	**0.047**
Common-law	25 (3.3)	17 (4.4)	
Widowed	92 (12.3)	64 (16.7)	
Separated or divorced	61 (8.2)	42 (10.9)	
Single, never married	61 (8.2)	34 (8.9)	
Country of birth*, n* (%)			
Canada	669 (89.9)	349 (91.1)	0.589
Other	75 (10.1) missing, *n =* 3	34 (8.9) missing, *n =* 1	
Language spoken most often at home, *n* (%)			
English	717 (96.0)	365 (95.1)	0.482
French	5 (0.7)	2 (0.5)	
Other	3 (0.4)	4 (1.0)	
More than one	22 (2.9)	13 (3.4)	
Employment status, *n* (%)			
Working full-time/part-time	203 (27.2)	135 (35.3)	**0.005**
Retired/unemployed/unable to work	542 (72.8) missing, *n =* 2	247 (64.7) missing, *n =* 2	
Household income, *n* (%)			
<$30 000	152 (26.3)	88 (31.4)	0.116
>$30 000	426 (73.7) missing, *n =* 169	192 (68.6) missing, *n =* 104	
(b)			
Self-perceived general health, *n* (%)			
Excellent/very good/good	647 (86.6)	303 (79.1)	**0.001**
Fair/poor	100 (13.4)	80 (20.9) missing, *n =* 1	
Have a regular medical doctor, *n* (%)			
Yes	729 (97.7)	370 (96.4)	0.183
No	17 (2.3) missing, *n =* 1	14 (3.6)	
Self-perceived oral health, *n* (%)			
Excellent/very good/good	620 (83.1)	296 (77.3)	**0.018**
Fair/poor	126 (16.9) missing, *n =* 1	87 (22.7) missing, *n =* 1	
Satisfaction with appearance of teeth, *n* (%)			
Very satisfied/satisfied	581 (77.9)	290 (75.7)	0.483
Neither satisfied nor dissatisfied	64 (8.6)	31 (8.1)	
Dissatisfied/very dissatisfied	101 (13.5) missing, *n =* 1	62 (16.2) missing, *n =* 1	
Frequency of dental visits, *n* (%)			
≥once per year	538 (73.0)	244 (64.4)	**0.003**
<once per year	199 (27.0) missing, *n =* 10	135 (35.6) missing, *n =* 5	
Avoided dental treatment of dental/oral problems in past year, *n* (%)			
Yes	113 (15.2)	74 (19.4)	0.069
No	632 (84.8) missing, *n =* 2	307 (80.6) missing, *n =* 3	
Dental insurance, *n* (%)			
Yes	366 (49.4)	202 (53.2)	0.233
No	375 (50.6) missing, *n =* 6	178 (46.8) missing, *n =* 4	

a Chi-square test unless otherwise noted.

b Standard deviation.

c *T*-test.

Bold indicates statistically significant.

**Table 3:** Forward stepwise logistic regression using the Wald statistic as the addition criteria (0 = completer, 1 = partial completer).

	*B*	Adjusted odds ratio	95% CI^a^	p Value^b^
Frequency of dental visits				
≥Once per year	0.36	1.44	1.03–2.00	**0.03**
Employment status				
Retired/unemployed/ unable to work	0.46	1.59	1.16–2.18	**<0.01**
Education				
Less than high school (reference group)		1.00		
Completed high school	0.33	1.39	0.85–2.26	0.19
Post secondary	0.61	1.83	1.30–2.59	**0.001**

B, logistic regression coefficient.

a Confidence interval.

b Criteria: entry *p* = 0.05, removal *p* = 0.10.

Bold indicates statistically significant.

## Discussion

Extensive surveys, such as the one described here, are vital to providing much needed oral health data, but they present a number of challenges and issues that are useful to describe here for future studies of this type.

### Challenges

#### Recruitment

Our survey response rate (completed cases/estimate of all eligible cases in the sample) was 21% for the phone interview and 13.5% for the clinical examination. The actual response rate may be higher, given our assumption that the proportion of eligible cases was the same for both the contacted and non-contacted sample^18^. Survey response rates in general have been declining over the past few decades^19^, with telephone surveys, in particular, experiencing significant declines in the last 10–15 years^18^,^20^. Response rates for surveys involving oral examinations are often low^21^–^23^, and comparisons between different studies are difficult to make, as many studies fail to report their response rates or do not clearly state how their response rates were calculated. In this case, the low response rate was a result of a high non-contact rate (39% of calls) as well as a high refusal rate (38% of eligible contacts). The latter may be a result of the requirement for the participant to agree to attend a clinical examination before the interview was conducted. For instance, 10% of eligible contacts indicated that they could not attend a clinical examination because they were physically disabled in some way. A significant number of people (34%) who agreed to participate and completed the telephone interview subsequently cancelled their examination appointment or simply failed to attend. Reasons given for cancellations were usually related to conflicting schedules or illness. A few people gave lack of transportation as a reason or said they had changed their mind about participating.

Recommended methods for improving these rates, such as call-backs for reaching non-contacts (up to three calls at varying times of the day) and using interviewers trained in reducing refusals^21^ were used in this survey. The implementation of the Canadian National ‘Do Not Call List’ just prior to the start of this study in 2008 may have had an impact on response rates (although research surveys are exempt from the registry); however, Link *et al.*^24^ found no significant impact on response rates after a similar registry was implemented in the USA.

In an effort to compensate for the anticipated low response rate, we overbooked clinical examinations by about 30%, extended the survey period by six additional sampling days and built oversampling into the study design.

#### Communications

Maintaining open and clear communication with all those involved in the research was another challenge encountered. This was exacerbated by using a telemarketing company located in another province to recruit participants. There were several instances where there was either a misunderstanding on the part of the participant or a miscommunication by the phone interviewer/recruiter with regard to the purpose of the study, what services were being offered or the location of the examination. When these issues were encountered by the survey team in the field, they were reported back to the project coordinator, who then addressed them with the telemarketing company. Participants were provided with a toll-free number to call the project coordinator directly, and this, no doubt helped to mitigate communication problems. Many people called the number to clarify the purpose of the study, to confirm their appointment time/location or to cancel/reschedule their appointment. Nonetheless, in two instances, people contacted their local police following contact by the telemarketing company, as they were concerned about telephone fraud. Law enforcement officials subsequently contacted the telemarketing company and the University to confirm the veracity of the study.

#### Space

Significant coordination was required to ensure space for the clinical examinations. Com-munity dental offices were used whenever possible. Many dentists volunteered the use of their private clinics on their day off or the use of an unused operatory. Where a dental clinic was not available, surveys were carried out using the portable dental chair and light in long-term care facilities, public health offices and small local hospitals.

### Analysis of non-response bias

Non-response bias is a concern in all surveys, as people who agree to participate may not be representative of the population being sampled. This may be an issue for surveys with high response rates as well as those with low response rates^21^, and a low response rate does not necessarily indicate non-response bias^25^,^26^. By comparing characteristics of all survey participants with census data of the target population, we obtained information about the differences between respondents and non-respondents, enabling us to adjust survey outcome data appropriately.

Our sample of people 65 years and older was comparable to the target population on all measures except level of education and country of birth. However, the sample of people 45–64 years old had more females, higher levels of education, lower levels of employment (more unemployed and those not in the labour force) and higher income levels than the target population. The differences in education and income indicate that the sample of people 45–64 years old might be biased towards a higher socio-economic status. There is a negative association between socio-economic status and oral health^27^,^28^, and therefore disease estimates on this sample may underestimate the true prevalence. Weighting and gender-related analyses will be used to correct prevalence rates.

Response rates (and possibly non-response bias) may be worsened when the survey requires participants to attend a clinical examination^21^,^29^. As described earlier, although participants in this study were required to agree to attend a clinical examination before being interviewed, 34% did not complete the examination. As socio-demographic, perceived general and oral health status, and oral health services utilisation data were collected through the telephone interview, this presented an opportunity to examine predictors of non-participation in clinical oral health surveys. Although completers and partial completers did not differ on important socio-demographic variables such as age, gender, rural/urban distribution or income levels, multivariate analysis revealed that being employed tended to decrease participant’s likelihood of attending the clinical examination, as did having less than high school education and having infrequent dental care. These results are supported by a previous oral health survey of adults aged 50 and more, where participants who completed only a phone interview were compared (using bivariate analyses) to those who completed both the interview and a clinical examination^22^. Although the percentage differences were small, the interview-only participants had lower levels of education and were more likely to be employed than those who completed both interview and examination. Unlike the present study, there were no significant differences between the two groups in frequency of dental visits. Similarly, Gilbert *et al*.^30^ found no association between time since last dental visit and participation in a clinical oral examination following a phone interview.

An understanding of the factors that make someone less inclined to participate in a clinical survey should help towards creating sample designs to overcome these barriers. In this study, most clinical examinations were conducted during daytime hours on weekdays. This may have biased the sample towards those who are not employed. Weekday daytime hours were chosen because of examiner and facility availability and because initial attempts to schedule evening appointments resulted in higher levels of no-shows than daytime appointments. It is possible that people who work simply have busier schedules in general (even outside work hours) than those who do not and were therefore unwilling to attend the appointment regardless of when it was scheduled. However, designing sampling times to be flexible may help to overcome this particular sampling bias.

Other factors such as education level and frequency of dental visits are more difficult to address. Education level may influence peoples’ perception of the importance of oral health and/or their perception of the importance of research. Frequency of dental visits may reflect one’s comfort level with visiting a dentist as well as one’s perception of the importance of oral health. In either case, clear explanation of the importance of the research and perhaps some incentive to attend the clinical examination might partially address this sampling bias.

## Conclusion

This study is the first province-wide assessment of oral health status of older adults in Canada. The survey will provide essential baseline information for future provincial oral health surveys and allow comparisons with national and international oral health data. Challenges encountered with recruitment, communication and logistics provide valuable lessons for future studies. Response rates were low, but are comparable with similar studies^22^,^23^, and any sampling bias is likely to cause our estimates of oral disease states to be conservative. Identification of employment, education and frequency of dental visits as predictors of survey participation and completion provides insight into possible non-response bias and suggests the potential for underestimation of the prevalence of oral diseases in this and similar surveys. This potential must be considered in analysis and in future recruitment strategies.
